# Supplementary material for: Transcranial focused ultrasound to V5 enhances human visual motion brain-computer interface by modulating feature-based attention
Source: Nat Commun. 2024 Jun 11;15:4382. doi: 10.1038/s41467-024-48576-8 (PMC11167030; doi:10.1038/s41467-024-48576-8)
Supplement: Supplementary file 1 — Supplementary Information [file 41467_2024_48576_MOESM1_ESM.pdf]

Supplementary Information

of

Transcranial Focused Ultrasound to V5 Enhances Human Visual Motion Brain-Computer Interface by Modulating Feature-Based Attention

Joshua Kosnoff[1], Kai Yu[1], Chang Liu[1,2], Bin He[1,3,*]

[1] Department of Biomedical Engineering, Carnegie Mellon University, Pittsburgh, PA 15237

[2] Department of Biomedical Engineering, Boston University, Boston, MA 02215

[3] Neuroscience Institute, Carnegie Mellon University, Pittsburgh, PA 15237

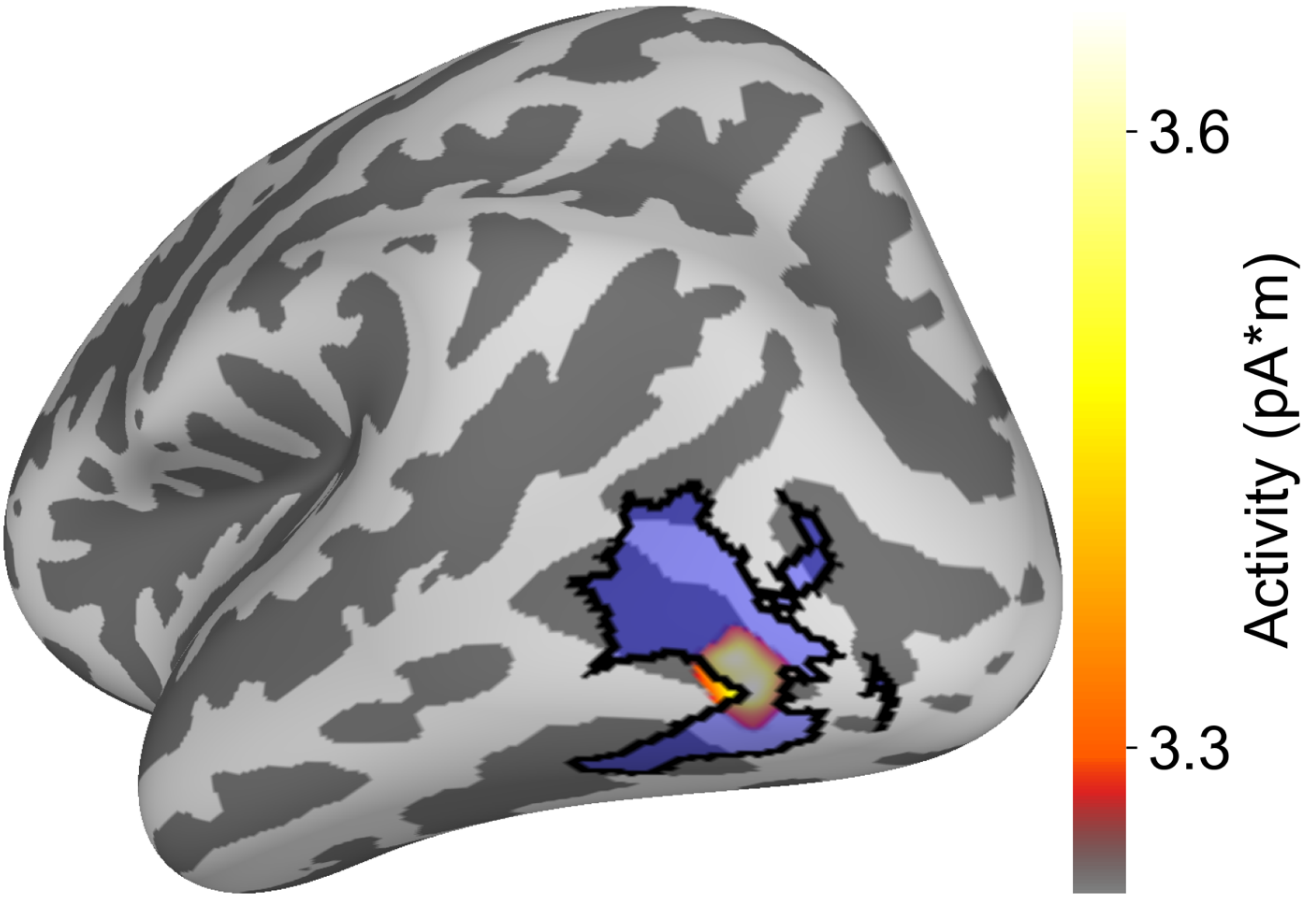

Supplemental Figure S1: The functional activity evoked by the mVEP BCI speller is located in the geometric center of V5, related to Methods and Results. EEG data from each subject's non-modulated training session were projected onto their cortical surface using EEG source imaging through minimum norm estimation. Data were bandpass filtered from 1 to 40 Hz, and the mean N200 activity (100 – 250 ms) was captured. Subsequently, the data were source morphed to a common FreeSurfer *FSAverage* brain[133] and averaged across all subjects (*N* = 24). The resulting data indicate that the cortical activation associated with the BCI task was in the geometric center of V5, outlined in black and highlighted in blue. This alignment corresponds to the targeted region of tFUS-GC.

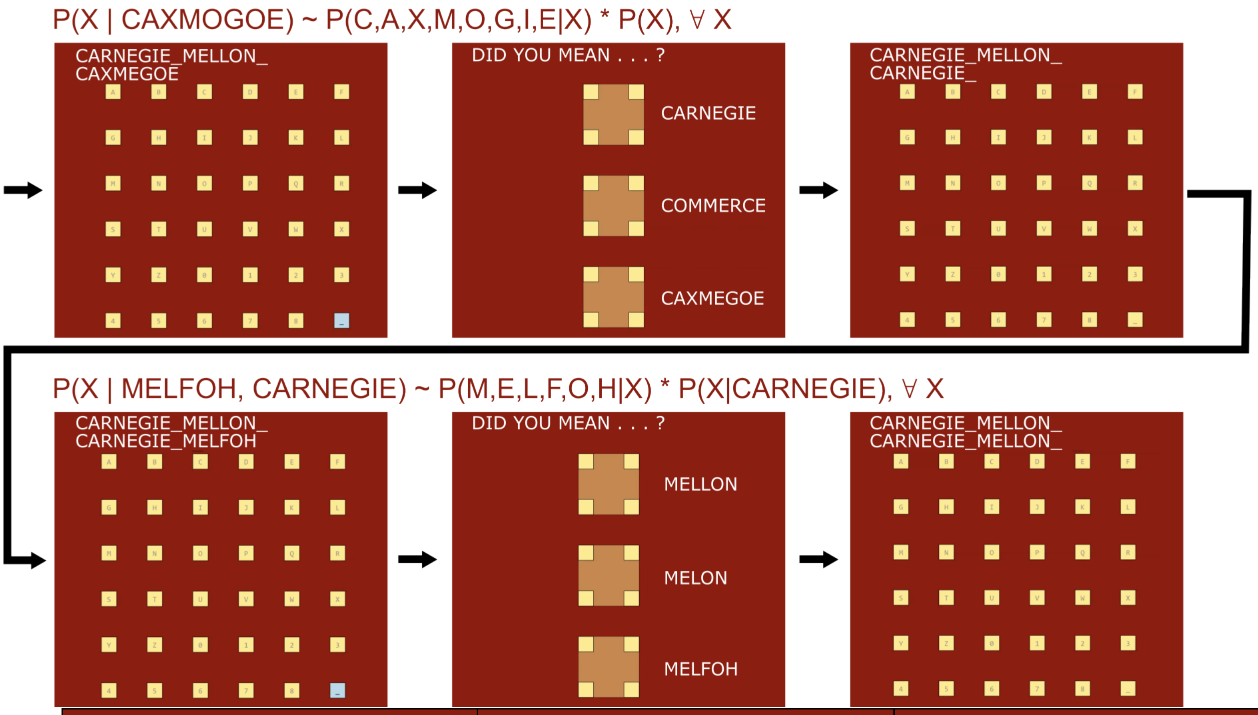

Typed Letters	Original Typed Accuracy	Online SCRIBE Result
CARNEGCE MAJLMN	71.4%	CARNEGIE MELLON
CARNAGIE MBLLON	85.7%	CARNEGIE MELLON
CARNEGGW MEIL0H	64.3%	CARNEGIE MELLON
CARNWLID MELXOM	71.4%	CARNEGIE MELLON
CAQOEG0E KE3WOC	50.0%	CARNEGIE MELLON
CA_NEAOE MGRLTN	57.1%	CARNEGIE MELLON
CAXMEGOE MELFOH	64.3%	CARNEGIE MELLON
CALNEAIE QELLON	78.6%	CARNEGIE MELLON
CARNDAIK AELLOH	71.4%	CARNEGIE MELLON
CARN2GLE MECLON	78.6%	CARNEGIE MELLON

Supplemental Figure S2. The Shared Control autoRegressive Integrated Bayesian Estimator (SCRIBE) can improve BCI speller performance by applying linguistic rules, related to Results. (top) Diagram of SCRIBE algorithm based on an online test (Supplementary Video 1 bottom right). At the end of every word, a greedy search is performed based on the typed letters and previous words to determine the most probable corrections (Equation 16). If there is no previous word, the probability of the singular word occurring is considered instead. The two most probable words and the originally typed letters are presented as correction options. When the user makes a selection, the previous word is updated to reflect the choice. (bottom) Selected examples of online SCRIBE results. Even when the user's actual accuracy is 50%, the words are still able to be corrected by considering a combination of Euclidean errors and linguistic patterns.

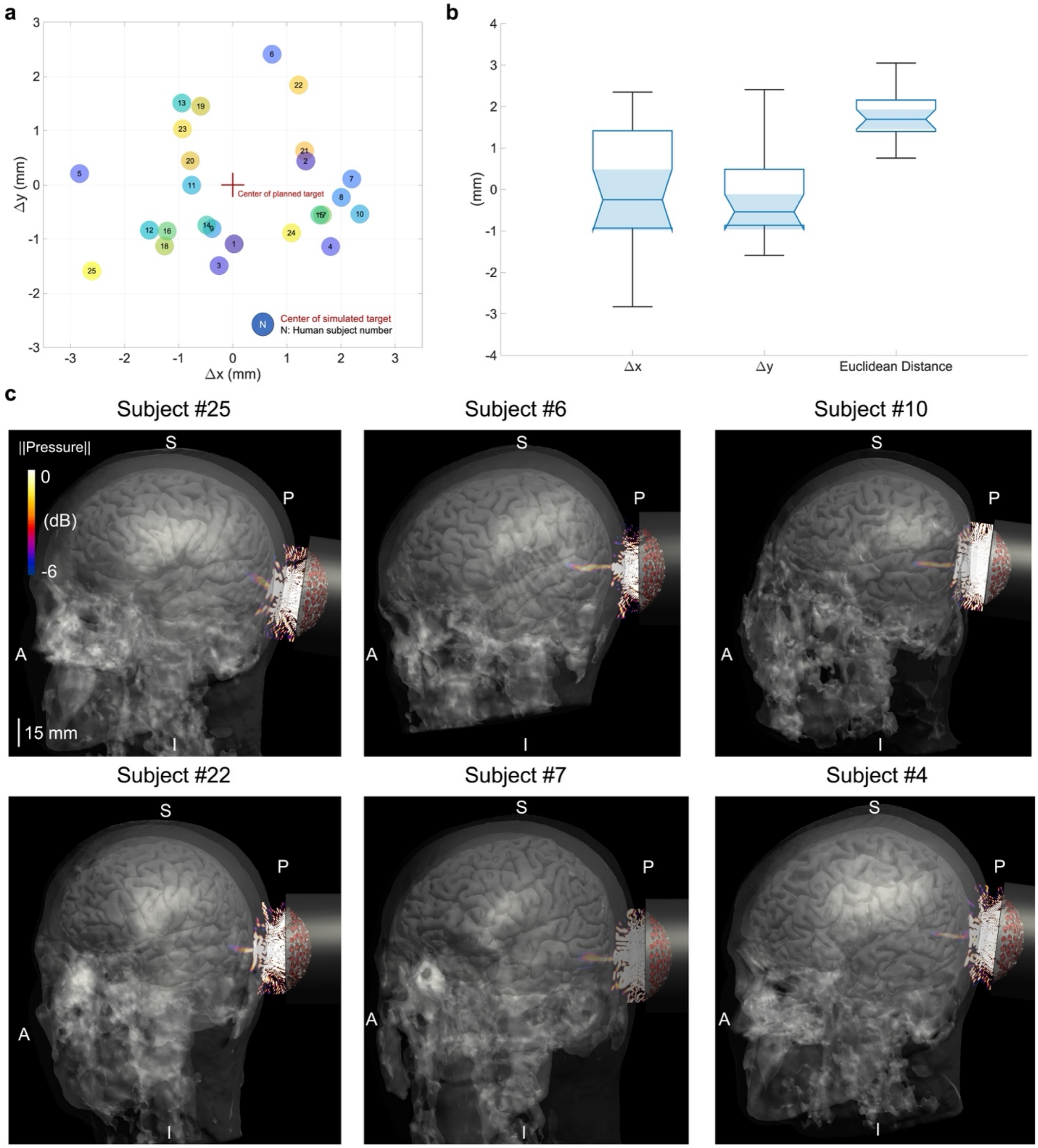

Supplemental Figure S3. tFUS simulations using subject-specific pseudo-CT-based skull models on each of 25 human subjects, related to Results and Figure 5. a) At the skull-brain interface normal to the sonication incidence, deflections are quantified using Δx and Δy relative to the center of planned target (shown as a red cross), while the colored disks scattered around the center of planned target represent the centers of simulated targets on individual human subjects. The subject number is labeled at the center of each disk. The center of the planned target is employed for brain navigation by choosing the geometrical

center of segmented V5 cortical brain. b) A box plot showing the deflection data distribution of all 25 human subjects in terms of Δx, Δy and Euclidean distance. For Δx, mean: 0.11 mm, median: -0.25 mm, standard deviation: 1.49 mm. For Δy, mean: -0.10 mm, median: -0.54 mm, standard deviation: 1.08 mm. For Euclidean distance, mean: 1.71 mm, median: 1.69 mm, standard deviation: 0.60 mm. c) In addition to the computer simulation on a representative individual (Subject #5) in Fig. 5, we presented the worst 6 cases of tFUS beam distortions in terms of the largest Euclidean distance from the center of the planned target to the center of each simulated target (the location of spatial peak pressure at the skull-brain interface). For instance, the simulation on Subject #25 demonstrates the worst deflection of 3.05 mm (Euclidean distance) away from the center of the planned target at V5. Overall, in these illustrated cases, the ultrasound focal beams still reside within the V5 region based on the individual anatomical segmentation.

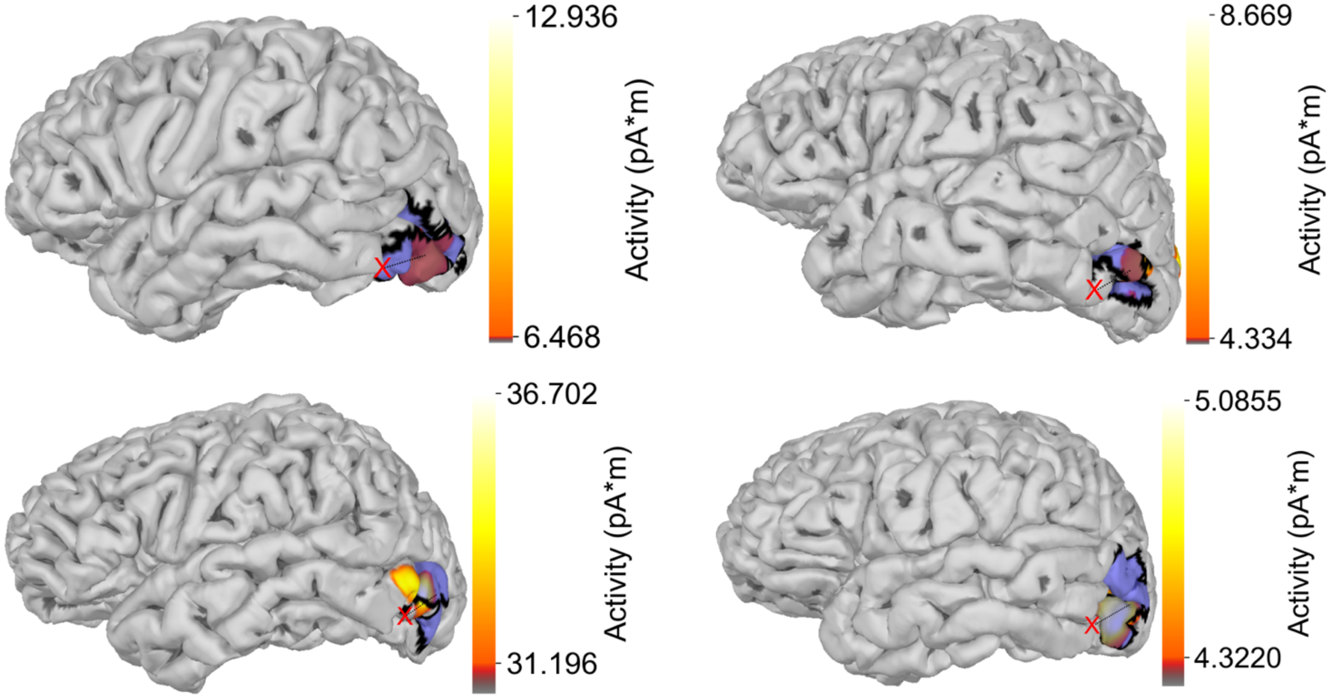

Supplemental Figure S4: Subject-specific targeting maps for tFUS-GP. The N200 timeframe was imaged for four subjects during the non-modulated BCI task. Area V5 is highlighted in purple and outlined in black. The reconstructed EEG source activities are colored from red to yellow-white. A 1.41 cm black dashed line (corresponding to the steering length of tFUS-GP) is started at the approximate geometric center of V5. The other end of the dashed line, marked with a red *X*, corresponds to the target location of tFUS-GP. In all cases, tFUS-GP is applied to the structural periphery of area V5, but beyond the main functional area.

Supplemental Table S1. Subject-wise characterization of transcranial ultrasound focus

Subject ID	*ΔZ* (mm)	*Pressure ratio*	$I_{SPTA.3}$ (mW/cm^2)	$I_{SPPA.3}$ (W/cm^2)
**1**	1.93	0.14	185.41	0.31
**2**	11.26	0.08	60.78	0.10
**3**	2.15	0.15	221.24	0.37
**4**	11.56	0.11	117.52	0.20
**5**	6.85	0.17	260.14	0.43
**6**	1.90	0.17	259.74	0.43
**7**	0.84	0.11	115.35	0.19
**8**	8.54	0.08	60.20	0.10
**9**	7.22	0.18	302.08	0.50
**10**	0.70	0.06	39.23	0.07
**11**	2.91	0.10	89.36	0.15
**12**	1.75	0.24	530.16	0.88
**13**	1.27	0.15	224.10	0.37
**14**	5.77	0.14	185.00	0.31
**15**	10.87	0.26	619.17	1.03
**16**	3.24	0.25	576.74	0.96
**17**	4.20	0.18	292.25	0.49
**18**	4.97	0.12	123.67	0.21
**19**	1.43	0.14	187.63	0.31
**20**	8.41	0.15	210.97	0.35
**21**	10.38	0.07	48.27	0.08
**22**	0.57	0.14	181.40	0.30
**23**	1.37	0.14	197.47	0.33
**24**	4.09	0.11	116.30	0.19
**25**	2.10	0.14	197.42	0.33

Note: *ΔZ* refers to axial shift of transcranial ultrasound focus (maximal ultrasound pressure magnitude in the brain) from the skull-brain interface; *Pressure ratio* is determined by dividing the maximum pressure within the brain by the maximum pressure in the head (including scalp, skull and brain). $I_{SPTA.3}$ is the derated spatial-peak temporal-average intensity, and $I_{SPPA.3}$ is the derated spatial-peak pulse-average intensity.

Supplemental Table S2. Validation analysis using double MAD outlier rejection

Data	Test	One-tailed *p*-value
	tFUS-GC < Non-Modulated	0.0067
**BCI Error**	tFUS-GC < Decoupled-Sham	0.0431
	tFUS-GC < tFUS-GP	0.0248
	tFUS-GC > Non-Modulated	<0.0001
**V5 Theta Power**	tFUS-GC > Decoupled-Sham	<0.0001
	tFUS-GC > tFUS-GP	0.0351
	tFUS-GC > Non-Modulated	<0.0001
**V5 Alpha Power**	tFUS-GC > Decoupled-Sham	<0.0001
	tFUS-GC > tFUS-GP	0.0242
	tFUS-GC > Non-Modulated	<0.0001
**SP Theta Power**	tFUS-GC > Decoupled-Sham	<0.0001
	tFUS-GC > tFUS-GP	<0.0001
	tFUS-GC > Non-Modulated	0.0036
**SP Alpha Power**	tFUS-GC > Decoupled-Sham	0.0036
	tFUS-GC > tFUS-GP	0.0157
**V5-IT Theta Correlation**	tFUS-GC < Non-Modulated	0.0024
**V5-IT Alpha Correlation**	tFUS-GC < Non-Modulated	0.0149
**V5-SP Theta Correlation**	tFUS-GC < Non-Modulated	0.1256
**V5-SP Alpha Correlation**	tFUS-GC < Non-Modulated	0.14

Note: Analysis for all major results were re-run using double median absolute deviation (double MAD) outlier rejection with a detection threshold of 3.5 double MAD distance from the median. All results are consistent with those in the main manuscript calculated with outlier rejection via interquartile range detection. Tests consisted of one-tailed z-tests based on linear mixed effect modeling, and specifics are specified in the main manuscript's Methods section.

Supplemental Table S3. 95% Confidence Intervals of bootstrapped linear mixed effect models (LMMs).

Effect	Estimate	95% CI Lower Bound	95% CI Upper Bound
**tFUS-GC on Euclidean Error**	-3.64	-6.31	-1.02
**tFUS-GC on V5 Theta Power**	2.33	1.9	2.76
**tFUS-GC on SP Theta Power**	1.44	0.939	1.9
**tFUS-GC on V5 Alpha Power**	1.33	1.01	1.62
**tFUS-GC on SP Alpha Power**	0.59	0.242	0.929
**tFUS-GC on V5-IT Theta Correlation**	-0.0000728	-0.0195	0.0195
**Non-Modulated V5-IT Theta Correlation**	0.0287	0.00944	0.0477
**tFUS-GC on V5-IT Alpha Correlation**	-0.000381	-0.0185	0.0192
**Non-Modulated V5-IT Alpha Correlation**	0.0205	0.00241	0.0396

The residuals of the linear mixed effect models were not normally distributed. While there have been multiple studies that demonstrate LMMs are robust even in such scenarios[125, 126], we performed case bootstrap analysis with 1,000 samples[127] to verify this for our data. Bootstrap resampling was implemented at the observational level, meaning each specific trial of data was be considered a case that could sampled. The 95% confidence intervals (CIs) for all of the models except those dealing with correlation resulted in strictly non-zero intervals for tFUS-GC, indicating that the factor itself is significant. For the correlation analysis, while the tFUS-GC CIs did contain zeros, the CIs were not overlapping with the CIs corresponding to their respective non-modulated condition. This is in line with our model results, as tFUS-GC did not result in significantly different correlation from all the control conditions, so tFUS-GC's effect on correlation may not be recognizable as a solely significant factor in-and-of itself, but it was still significantly different from the non-modulated experimental condition.
