# Supplementary material for: Transcranial focused ultrasound to V5 enhances human visual motion brain-computer interface by modulating feature-based attention
Source: Nat Commun. 2024 Jun 11;15:4382. doi: 10.1038/s41467-024-48576-8 (PMC11167030; doi:10.1038/s41467-024-48576-8)
Supplement: Supplementary file 3 — Description of Additional Supplementary Files [file 41467_2024_48576_MOESM3_ESM.pdf]

Description of Additional Supplementary Files

Supplemental Video 1. Four online examples of SCRIBE allowing two subjects to fully correct their previous spelling mistakes. (top left) Subject 1 originally typed “CARN2GLE MECLON” (original accuracy = 78.6%). (top right) Subject 1 originally typed “CALNEAIE QELLON” (original accuracy = 78.6%). (bottom left) Subject 1 originally typed “CARNDAIK AELLOH” (original accuracy = 71.4%). (bottom right) Subject 2 originally typed “CAXMEGOE MELFOH” (original accuracy = 64.3%). SCRIBE performed a greedy search over all words in a dictionary and calculated the probability of each word being the user’s intention as a function of Euclidean error between the typed letters and the word, as well as the probability of the word occurring given the previous word in the sequence based on Google N Gram’s 2018 word frequency data. In all four cases, SCRIBE calculated the probability of “CARNEGIE” as one of two most probable word choices for the first set of letters and presented it as an option to the user. The users were able to correctly select the option, and their typos were corrected. SCRIBE’s word sequence database was internally updated with the word. Then, SCRIBE calculated MELLON as one of the two most probable choices given the second set of letters the users typed and probability of the word following the previous word (“CARNEGIE”).

Supplemental Video 2. An additional online example of SCRIBE from a third subject. The subject originally typed “CARNEGGW MEIL0H” (64.3% raw accuracy), but with the help of SCRIBE, was able to fully correct the output to “CARNEGIE MELLON
