# Supplementary material for: Transcranial focused ultrasound to V5 enhances human visual motion brain-computer interface by modulating feature-based attention
Source: Nat Commun. 2024 Jun 11;15:4382. doi: 10.1038/s41467-024-48576-8 (PMC11167030; doi:10.1038/s41467-024-48576-8)
Supplement: Supplementary file 6 — Reporting Summary [file 41467_2024_48576_MOESM6_ESM.pdf]

nature portfolio

Corresponding author(s): Bin He

Last updated by author(s): Apr 18, 2024

Reporting Summary

Nature Portfolio wishes to improve the reproducibility of the work that we publish. This form provides structure for consistency and transparency in reporting. For further information on Nature Portfolio policies, see our Editorial Policies and the Editorial Policy Checklist.

Statistics

For all statistical analyses, confirm that the following items are present in the figure legend, table legend, main text, or Methods section.

n/a	Confirmed	
☐	☒	The exact sample size (*n*) for each experimental group/condition, given as a discrete number and unit of measurement
☐	☒	A statement on whether measurements were taken from distinct samples or whether the same sample was measured repeatedly
☐	☒	The statistical test(s) used AND whether they are one- or two-sided<br>*Only common tests should be described solely by name; describe more complex techniques in the Methods section.*
☐	☒	A description of all covariates tested
☐	☒	A description of any assumptions or corrections, such as tests of normality and adjustment for multiple comparisons
☐	☒	A full description of the statistical parameters including central tendency (e.g. means) or other basic estimates (e.g. regression coefficient) AND variation (e.g. standard deviation) or associated estimates of uncertainty (e.g. confidence intervals)
☐	☒	For null hypothesis testing, the test statistic (e.g. *F*, *t*, *r*) with confidence intervals, effect sizes, degrees of freedom and *P* value noted<br>*Give P values as exact values whenever suitable.*
☐	☒	For Bayesian analysis, information on the choice of priors and Markov chain Monte Carlo settings
☒	☐	For hierarchical and complex designs, identification of the appropriate level for tests and full reporting of outcomes
☐	☒	Estimates of effect sizes (e.g. Cohen's *d*, Pearson's *r*), indicating how they were calculated

Our web collection on statistics for biologists contains articles on many of the points above.

Software and code

Policy information about availability of computer code

Data collection	EEG Data were collected with BrainVision Recorder version 1.21.0004
Data analysis	Analysis scripts were made using Python 3.7 and the following packages:<br>matplotlib==3.7.0<br>mne==1.4.2<br>numpy==1.23.5<br>pandas==1.5.3<br>scipy==1.10.0<br>seaborn==0.12.2<br>statsmodels==0.13.5<br>autoreject==0.4.2<br><br>Additional analysis was also performed in R using:<br>lme4<br>multcomp<br>BayesFactor<br>lmeresampler<br><br>Code for both programs are uploaded to a GitHub Repository

For manuscripts utilizing custom algorithms or software that are central to the research but not yet described in published literature, software must be made available to editors and reviewers. We strongly encourage code deposition in a community repository (e.g. GitHub). See the Nature Portfolio guidelines for submitting code & software for further information.

Data

Policy information about availability of data

All manuscripts must include a data availability statement. This statement should provide the following information, where applicable:

- Accession codes, unique identifiers, or web links for publicly available datasets
- A description of any restrictions on data availability
- For clinical datasets or third party data, please ensure that the statement adheres to our policy

The data are presented in the paper and supplementary information. Additional data and codes are made public in Figshare and GitHub.

Research involving human participants, their data, or biological material

Policy information about studies with human participants or human data. See also policy information about sex, gender (identity/presentation), and sexual orientation and race, ethnicity and racism.

Reporting on sex and gender	We obtained and reported subject sex. We did not collect information on subject gender.
Reporting on race, ethnicity, or other socially relevant groupings	We did not obtain or report information regarding race, ethnicity, or other socially relevant groupings.
Population characteristics	Our study participants were young and healthy individuals (13 male / 12 female; mean age: 24.0 ± 5.59).
Recruitment	Our study is posted to ClinicalTrials.gov and our study fliers were also posted to numerous study-recruitment boards around the university area. Interested individuals called/emailed us and we performed a prestudy safety screening to exclude subjects with a family or personal history of epilepsy or fainting, as well as subjects with metallic implants who could not undergo MRIs.
Ethics oversight	Our study is reviewed and approved by Advarra Institutional Review Board.

Note that full information on the approval of the study protocol must also be provided in the manuscript.

Field-specific reporting

Please select the one below that is the best fit for your research. If you are not sure, read the appropriate sections before making your selection.

☒ Life sciences ☐ Behavioural & social sciences ☐ Ecological, evolutionary & environmental sciences

For a reference copy of the document with all sections, see nature.com/documents/nr-reporting-summary-flat.pdf

Life sciences study design

All studies must disclose on these points even when the disclosure is negative.

Sample size	We collected data from 25 human subjects. The sample size was chosen based on other high-impact tFUS neuromodulation studies (i.e. Legon et al 2014 N = 12 subjects; Yaakub et al 2023 N = 24 subjects)
Data exclusions	A subject's data were excluded from analysis if their non-modulated BCI classifier was random chance
Replication	The reproducibility of findings is in the averaging of data across our 25 human subjects.
Randomization	This study was a cross-over study, where multiple conditions were tested on each subject. Experiment condition order was randomized.
Blinding	Investigators were not blinded to group allocation. Blinding was not possible due to the need to manually target and activate the ultrasound transducer.

Reporting for specific materials, systems and methods

We require information from authors about some types of materials, experimental systems and methods used in many studies. Here, indicate whether each material, system or method listed is relevant to your study. If you are not sure if a list item applies to your research, read the appropriate section before selecting a response.

Materials & experimental systems

n/a	Involved in the study
☒	☐ Antibodies
☒	☐ Eukaryotic cell lines
☒	☐ Palaeontology and archaeology
☒	☐ Animals and other organisms
☒	☐ Clinical data
☒	☐ Dual use research of concern
☒	☐ Plants

Methods

n/a	Involved in the study
☒	☐ ChIP-seq
☒	☐ Flow cytometry
☐	☒ MRI-based neuroimaging

Plants

Seed stocks: N/A (No plants used in this study, but the plant section will not go away)

Novel plant genotypes: N/A

Authentication: N/A

Magnetic resonance imaging

Experimental design

Design type: Resting state structural brain scan

Design specifications: N/A (no experiments within the MRI machine)

Behavioral performance measures: N/A

Acquisition

Imaging type(s): Structural

Field strength: 3 Tesla

Sequence & imaging parameters:
Sequence Name: tfl3d1_16ns
Imaging Type: Whole brain
Slice Thickness = 1
Echo Numbers = 1
Echo Time = 2.9
Flip Angle = 8

Area of acquisition: Whole brain scan

Diffusion MRI: ☐ Used ☒ Not used

Preprocessing

Preprocessing software: Structural MRI was processed using FreeSurfer version 7 to obtain regions of interests (noted below). The structural MRI was also converted to pseudo-CT images using an open-source software package https://github.com/sitiny/mr-to-pct.

Normalization: N/A

Normalization template: N/A

Noise and artifact removal: N/A

Volume censoring: N/A

Statistical modeling & inference

Model type and settings	We used Linear Mixed Effect models. Experiment condition were fixed effects and subjects were random effect variables. In the behavioral test, we also considered experiment order and number of repeats as fixed effects.
Effect(s) tested	We tested EEG powers in different frequency bands, behavioral errors from the BCI, and Pearson's correlation coefficient differences.

Specify type of analysis: ☐ Whole brain ☒ ROI-based ☐ Both

Anatomical location(s)	We used Brodmann Area (BA_exvivo) atlas' left hemisphere V5 (MT_exvivo-lh) label and the Desikan-Killiany (aparc) atlas' parcellations for the left hemisphere superior parietal lobe (superiorparietal-lh) and the left hemisphere inferior temporal lobe (inferiortemporal-lh)
Statistic type for inference (See Eklund et al. 2016)	Statistical inference was not applied directly on MRI data. MRI and EEG together were used to perform EEG source imaging and analysis. Average ROI activity was analyzed across subjects / trials for power and correlation using t-tests on linear mixed effect models.
Correction	Both Bonferroni and FDR multiple comparison corrections were used, as noted in the manuscript. Correction was applied in R.

Models & analysis

n/a	Involved in the study
☐	☒ Functional and/or effective connectivity
☒	☐ Graph analysis
☒	☐ Multivariate modeling or predictive analysis

Functional and/or effective connectivity	Pearson's Correlation coefficient
